# Prevalence and Associated Factors of Sarcopenia in Patients With Diabetic Foot Ulcers: A Systematic Review and Meta‐Analysis

**DOI:** 10.1002/jfa2.70201

**Published:** 2026-09-16

**Authors:** Wang Hui, Yang Yaping, Lv Tan, Mao Zhigang, Dai Xinyi, Peng Yinbo, Wang Jing

**Affiliations:** ^1^ Department of Burn & Plastic and Reconstructive Surgery Shanghai Ninth People's Hospital Shanghai Jiaotong University School of Medicine Shanghai China; ^2^ Department of Nursing Yangpu Hospital School of Medicine Tongji University Shanghai China; ^3^ Yangpu Hospital School of Medicine Tongji University Shanghai China

**Keywords:** diabetic foot, influencing factors, prevalence, sarcopenia

## Abstract

**Objective:**

To systematically evaluate the prevalence and influencing factors of diabetic foot sarcopenia.

**Methods:**

We systematically searched PubMed, Embase, Scopus, Web of Science, the Cochrane Library, SinoMed, CNKI, Wanfang, and VIP databases from inception until June 10, 2025, for studies reporting the prevalence and/or influencing factors of sarcopenia in patients with diabetic foot. All meta‐analyses were performed using R software (version 4.4.2).

**Results:**

A total of 1164 patients with diabetic foot ulcers were included in 7 studies. Meta‐analysis showed that the prevalence of diabetic foot sarcopenia was 31% (95% CI: 0.22–0.41). Pooling of data from two studies indicated a significant association between longer diabetes duration and sarcopenia (OR = 3.33, 95% CI: 1.61–6.87, and *p* < 0.01). The evidence for other factors was insufficient for meta‐analysis.

**Conclusion:**

This review confirms a high prevalence of sarcopenia in patients with diabetic foot. Although an association with longer diabetes duration was observed, this is based on limited studies and requires further validation. These findings advocate for the consideration of early sarcopenia screening and preventive strategies within comprehensive diabetic foot care, potentially mitigating muscle loss and improving long‐term outcomes.

## 1 Introduction

Diabetic foot, as one of the most severe chronic complications of diabetes [1], has a global prevalence of up to 25%, leading to a significant decline in patients' quality of life and imposing a huge economic burden on healthcare—non‐traumatic amputations account for 40%–60% of diabetes‐related amputations [2, 3]. Recent studies have found that the presence of sarcopenia (characterized by progressive loss of skeletal muscle mass, strength decline, and/or physical function deterioration) may further worsen the prognosis for diabetic foot patients, significantly increasing the risk of amputation and all‐cause mortality [4]. The pathological mechanisms underlying this vicious cycle involve multiple factors: insulin resistance promotes muscle protein breakdown, a chronic inflammatory state (such as elevated IL‐6 and TNF‐α) accelerates muscle wasting, whereas the reduction in muscle mass exacerbates metabolic disorders by decreasing glucose disposal capability [5]. Notably, the unique pathophysiological changes in diabetic foot patients—which include muscle disuse due to long‐term reduced activity, tissue hypoxia caused by microcirculation disorders, and a persistent catabolic state induced by chronic wounds—may increase their risk of sarcopenia more than that of typical diabetes patients [6, 7]. Given this clinically underrecognized issue, the current study aims to achieve three key objectives through a meta‐analysis: (1) to accurately quantify the prevalence of sarcopenia in patients with diabetic foot; (2) to identify factors associated with sarcopenia in this population; and (3) to assess the methodological heterogeneity of existing studies, thus providing a theoretical basis for establishing an evidence‐based early screening system and multidisciplinary management strategies.

## 2 Methods

The preferred reporting items for systematic reviews and meta‐analyses (PRISMA) standards [8]were followed while reporting this systematic review. The protocol is registered in 2025 with the international prospective register of systematic reviews (PROSPERO) under the registration number: CRD420251084646.

### 2.1 Literature Search Strategy

We systematically searched CNKI, VIP, Wanfang Data, SinoMed, PubMed, Embase, Web of Science, Scopus, and the Cochrane Library databases for relevant Chinese and English literature published from inception until June 10, 2025. The search strategy combined subject headings with free‐text words, including but not limited to: “diabetic foot,” foot ulcer, “sarcopenia,” “Muscular Atrophy,” and “risk factors.” The complete search strategy is detailed in Supporting Information S1: Table S1.

### 2.2 Literature Inclusion and Exclusion Criteria

Inclusion criteria were as follows: ① the subjects of the study are patients diagnosed with diabetic foot, aged over 18; ② the study provided a precise diagnosis of sarcopenia; and ③ outcome indicators report the prevalence or influencing factors of sarcopenia in patients with diabetic foot. Exclusion criteria were as follows: ① animal research; ② conference papers, reviews, case reports, or duplicate publications; ③ research on non‐Chinese and English languages; and ④ low‐quality study.

### 2.3 Literature Screening and Data Extraction

Two researchers independently conducted literature screening, information extraction, and cross‐checking using NoteExpress 3.7. Any disagreements were resolved through consultation with a third reviewer. When information was incomplete, the corresponding authors were contacted via email for additional details. The screening process comprised a preliminary review of titles and abstracts, followed by a full‐text assessment of potentially relevant studies.

### 2.4 Literature Quality Assessment

The agency for healthcare research and quality (AHRQ) scale was used to evaluate the risk of bias in cross‐sectional studies, with a total score of 11 points. Evaluation responses were recorded as “yes”, “no”, or “unclear”; a “yes” response scored 1 point, whereas all other responses scored 0 points, with higher scores indicating better methodological quality. Scores of 8–11, 4–7, and 0–3 were classified as high, moderate, and low quality, respectively [9]. Cohort and case‐control studies were assessed using the Newcastle–Ottawa Scale (NOS) [10], which comprises three domains—study population selection, inter‐group comparability, and exposure/outcome assessment—with a total of eight items. The maximum score is 9 points, with scores of 0–3 indicating low quality, 4‐6 moderate quality, and ≥ 7 high quality. Two researchers independently performed the quality assessment, with any disagreements resolved through consultation with a third reviewer.

### 2.5 Statistical Analysis

All meta‐analyses were performed using R software (version 4.4.2). Odds ratios (OR) and their 95% confidence intervals (CI) were used as the effect measures. The Q‐test (*p*‐value) and *I* ^2^ statistics were used to assess heterogeneity among studies; if no significant heterogeneity was present (*I* ^2^ ≤ 50%, *p* > 0.10), a fixed‐effects model was applied; otherwise, a random‐effects model was used. Two researchers evaluated clinical and methodological heterogeneity based on study design, diagnostic criteria, measurement tools, and Wagner grade, and explored the sources of heterogeneity through subgroup analyses. Meta‐analyses were conducted for factors reported in two or more studies. Funnel plots and Egger's test were used to evaluate publication bias, with a significance level set at *α* = 0.05. Sensitivity analysis was performed using a leave‐one‐out method to verify the robustness of the results.

## 3 Results

### 3.1 Literature Search Results

A preliminary search of the literature databases yielded 295 records (63 in Chinese and 232 in English). After a stepwise screening process, 7 studies were finally included, encompassing 1164 patients with diabetic foot, of whom 359 were diagnosed with sarcopenia [11, 12, 13, 14, 15, 16, 17]. The literature retrieval and screening process are shown in Table 1.

**TABLE 1:** Flow chart of literature retrieval.

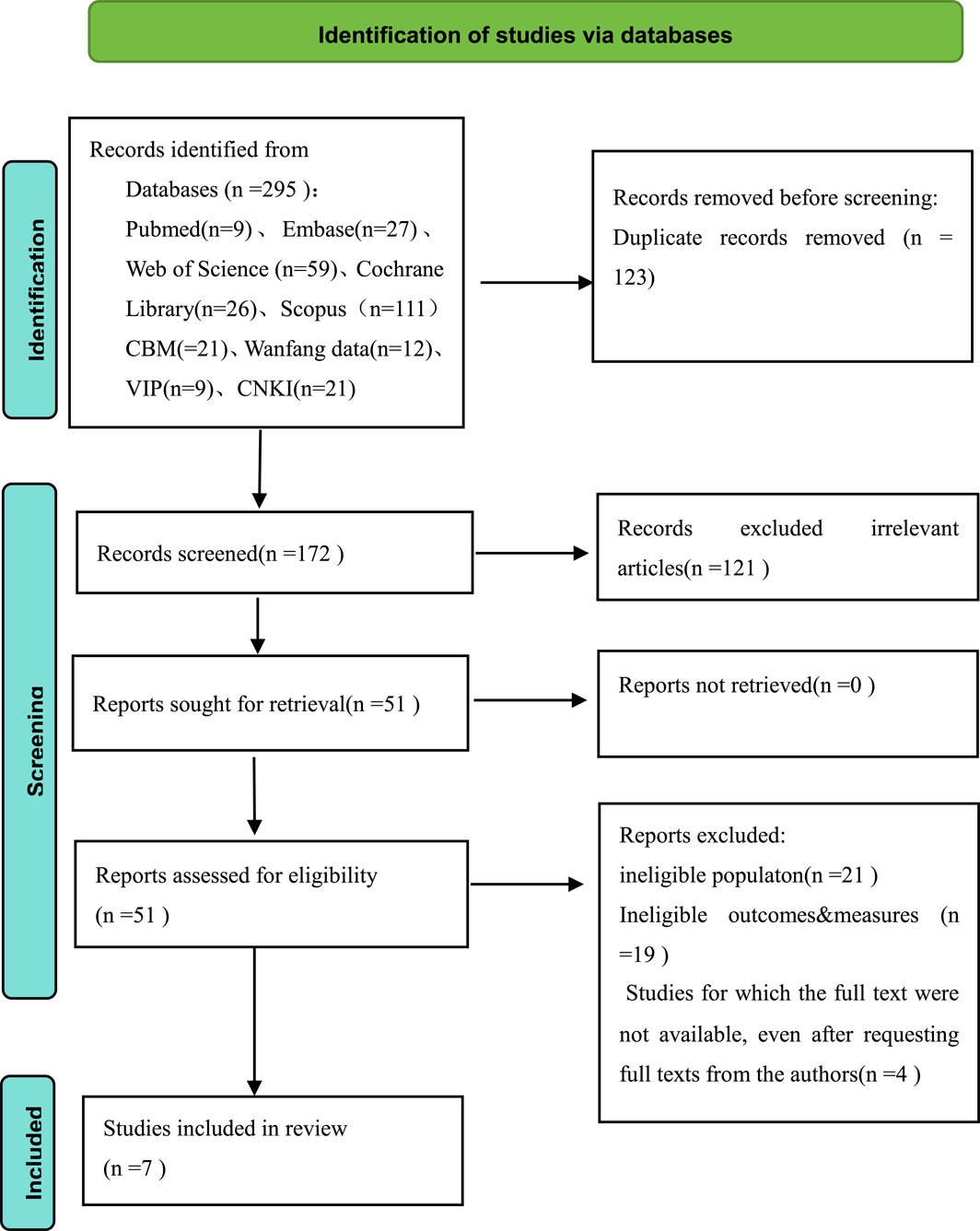

### 3.2 Basic Characteristics and Quality Evaluation of Included Literature

This study ultimately included 7 articles, all of which were cross‐sectional study designs and published after 2017. It used the 2019 Asian Working Group on Sarcopenia (AWGS) [18], 2014 Asian Working Group on Sarcopenia (AWGS) [19], 2019 European Working Group on sarcopenia for the Elderly (EWGSOP) [20], or Baumgartner's diagnostic criteria [21] as diagnostic criteria for sarcopenia and measured their muscle mass using CT, DXA, or BIA. These 7 articles mentioned a total of 15 influencing factors. The basic characteristics of this study are shown in Table 2.

**TABLE 2:** Summary characteristics of included studies.

Author (year)	Age (years)/mean (SD) or range	Country	Diagnostic criteria for sarcopenia	Muscle measurement tool	Staging of diabetic foot	Sample size (male/female)	Outcome	Influencing factors	AHRQ
Bai et al. (2023) [11]	—	China	2014 AWGS	—	—	80 (43/37)	^a^ ^,^ ^b^	①②③④⑤	5
Kuang et al. (2023) [12]	63.61 ± 9.32	China	2019 AWGS	DXA	Wagner 1‐5	108 (72/36)	^a^ ^,^ ^b^	⑥⑦⑧	6
Ni et al. (2023) [13]	66.59 ± 10.21	China	Baumgartner	DXA	—	255 (177/78)	^a^	—	6
Yang et al. (2020) [14]	66.42 ± 11.07	China	Baumgartner	DXA	—	257 (155/102)	^a^ ^,^ ^b^	⑨⑩⑪	6
Lin et al. (2017) [15]	76.70 ± 8.87	China	2014 AWGS	BIA	Wagner 2	273 (135/138)	^a^ ^,^ ^b^	①④⑦⑨⑫⑮	6
Cao et al. (2018) [16]	66.84 ± 11.18	China	2014 AWGS	DXA	Wagner 1–5	120 (67/53)	^a^	—	6
Wang et al. (2025) [17]	58.59 ± 8.31	China	2019 EWGSOP	CT	Sinbad 0‐6	71 (58/13)	^a^ ^,^ ^b^	⑬⑭	7

*Note:* ① Glycosylated hemoglobin(HbA1c); ② vitamin D deficiency; ③ protein intake; ④ long‐standing diabetes; ⑤ reduced exercise; ⑥ BMI; ⑦ transcutaneous oxygen partial pressure(TcPO2); ⑧ C‐reactive protein(crp); ⑨ age; ⑩ gender; ⑪ neuropathy symptom score; ⑫ ankle brachial index(ABI); ⑬ Sinbad system score; ⑭ wound pH value; ⑮ nerve conduction velocity.

^a^ I, prevalence.

^b^ influence factor.

### 3.3 Meta‐Analysis Results

#### 3.3.1 Overall Prevalence of Diabetic Foot Sarcopenia

**FIGURE 1:**
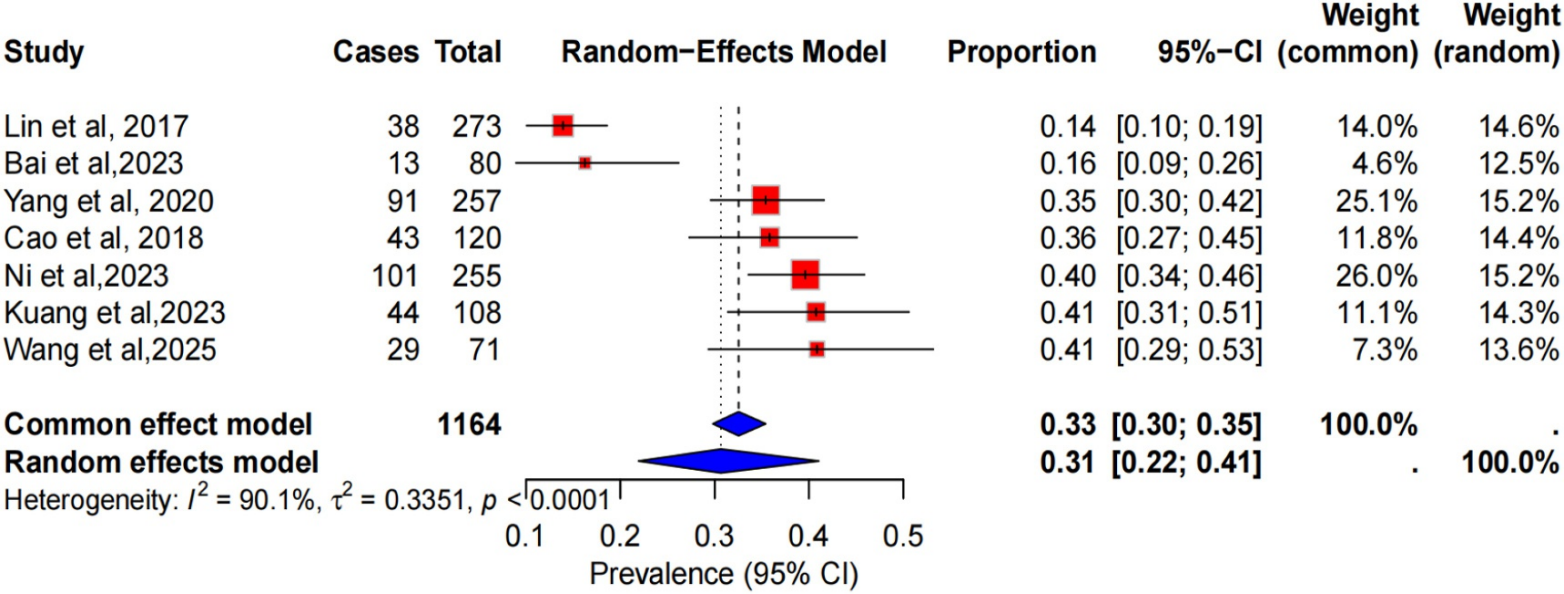
Meta‐analysis results of overall prevalence.

In the 7 studies included, the prevalence of sarcopenia in patients with diabetic foot ranged from 14% to 41%. The analysis using a random effects model showed that the overall prevalence of sarcopenia in this patient population was 31% (95% CI: 0.22–0.41, *I* ^2^ > 50%, and *p* < 0.001), as shown in Figure 1.

#### 3.3.2 Subgroup Analysis of the Prevalence of Diabetic Foot Sarcopenia

A meta‐analysis was conducted on the prevalence of sarcopenia based on diagnostic criteria, muscle mass measurement tools, and Wagner grade. Based on different diagnostic criteria for sarcopenia, there was a significant difference in prevalence (*I* ^2^ = 90.1%, *p* < 0.001); using the 2014 AWGS criteria, the prevalence was 21% (95% CI: 0.12–0.33, *I* ^2^ = 91.8%, and *p* < 0.001), and it was 38% (95% CI: 0.33–0.42, *I* ^2^ = 0%, *p* > 0.05) according to the Baumgartner method. According to different tools for measuring muscle mass, there was significant heterogeneity in prevalence (*I* ^2^ = 90.1%, *p* < 0.001); the prevalence detected by DXA was 38% (95% CI: 0.34–0.41, *I* ^2^ = 0%, *p* > 0.05) and by CT was 41% (95% CI: 0.29–0.53), both significantly higher than the 14% (95% CI: 0.10–0.19) of BIA detection. There are significant differences in the prevalence rates across different Wagner grades (*p* < 0.001); the prevalence of grade 1–2 was 23% (95% CI: 0.13–0.37), which was significantly lower than the prevalence of grade 3–4 at 48% (95% CI: 0.38–0.59, *I* ^2^ = 3.5, *p* > 0.05), whereas the prevalence of grade 5 was 90% (95% CI: 0.53–0.99, *I* ^2^ = 0, *p* > 0.05), which was significantly higher than that of grade 1–2 and 3–4. Specific results are shown in Table 3.

**TABLE 3:** Subgroup analysis of the prevalence of sarcopenia in diabetic foot patients.

Subgroups	Number of included studies	Heterogeneity test		Meta‐analysis related results		
		*I* ^2^ (%)	*p*	Prevalence rate (%)	Prevalence (95% CI)	*p*
Diagnostic criteria						0.01
2014 AWGS	3	91.8	0.01	0.21	(0.12, 0.33)	
2019 AWGS	1	—	—	0.41	(0.31, 0.51)	
2019 EWGSOP	1	—	—	0.41	(0.29, 0.53)	
Baumgartner	2	0	0.33	0.38	(0.33, 0.42)	
Tools						0.01
BIA	1	—	—	0.14	(0.10, 0.19)	
DXA	4	0	0.67	0.38	(0.34, 0.41)	
CT	1	—	—	0.41	(0.29, 0.53)	
Wagner grade						0.01
Grade 0	0	—	—	—		
Grade 1–2	3	87.3	0.01	0.23	(0.13, 0.37)	
Grade 3–4	2	3.5	0.31	0.48	(0.38, 0.59)	
Grade 5	2	0	0.61	0.90	(0.53, 0.99)	

*Note:* Wagner grade 0, with risk factors for foot ulcers, currently no ulcers; grade 1, clinically no infection, but superficial ulcers present on the skin; grade 2, no abscess or bone infection, ulcer extending to ligaments, tendons, and joints; grade 3, ulcers associated with osteomyelitis and deep abscesses; grade 4, localized gangrene (located in the toes, heel, or forefoot); grade 5, gangrene of the entire foot.

#### 3.3.3 Sensitivity Analysis and Publication Bias Analysis

**FIGURE 2:**
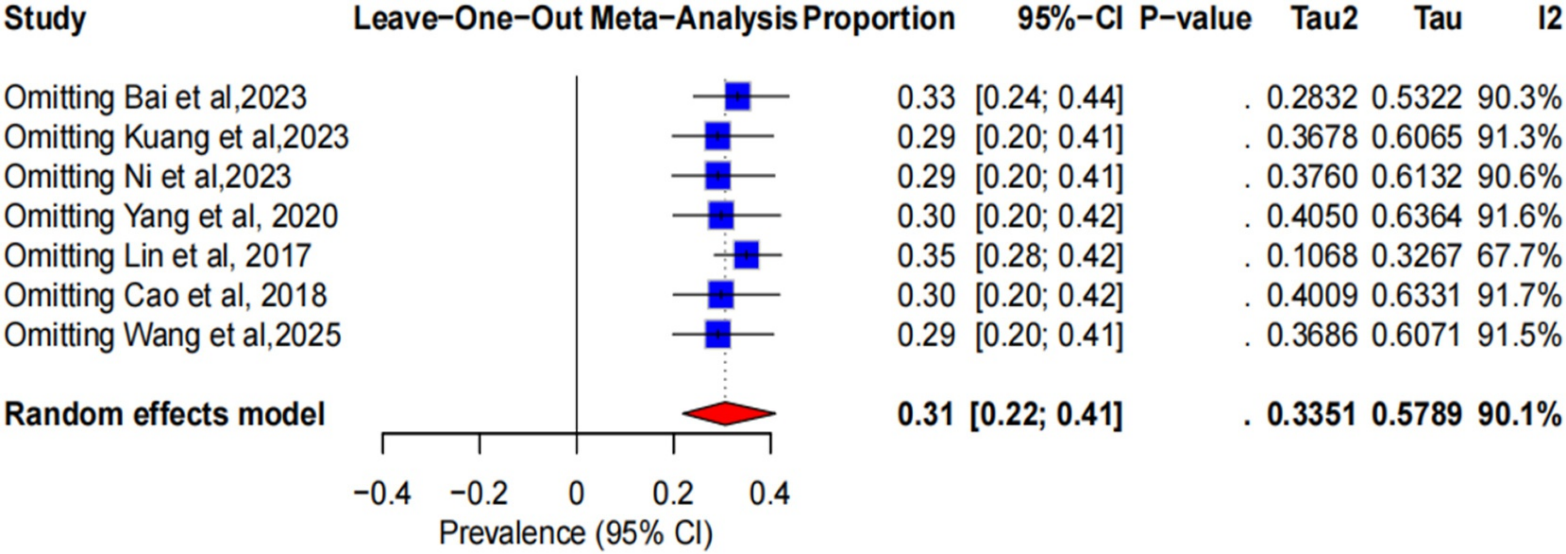
Leave‐one‐out forest plot.

The combined prevalence of sarcopenia in patients with diabetic foot was 31% (95% CI: 0.22–0.41), with high heterogeneity (*I* ^2^ = 90.1% ad *p* < 0.001). After sequentially removing each study, the range of combined prevalence varied from 29% to 35.0%, with no extreme fluctuations. After removing Lin et al. 2017 [15], the combined prevalence significantly increased to 35.0% (95% CI: 0.28–0.42), and the heterogeneity significantly decreased (*I* ^2^ dropped from 90.1% to 67.7%), indicating that this study was one of the main sources of heterogeneity (Figure 2). The fixed effects model estimates a prevalence rate of 32.5% [95% CI: 0.30–0.35], which is less than 5% different from the random effects model's estimated prevalence rate of 31% (95% CI: 0.22–0.41), supporting the robustness of the results.

**FIGURE 3:**
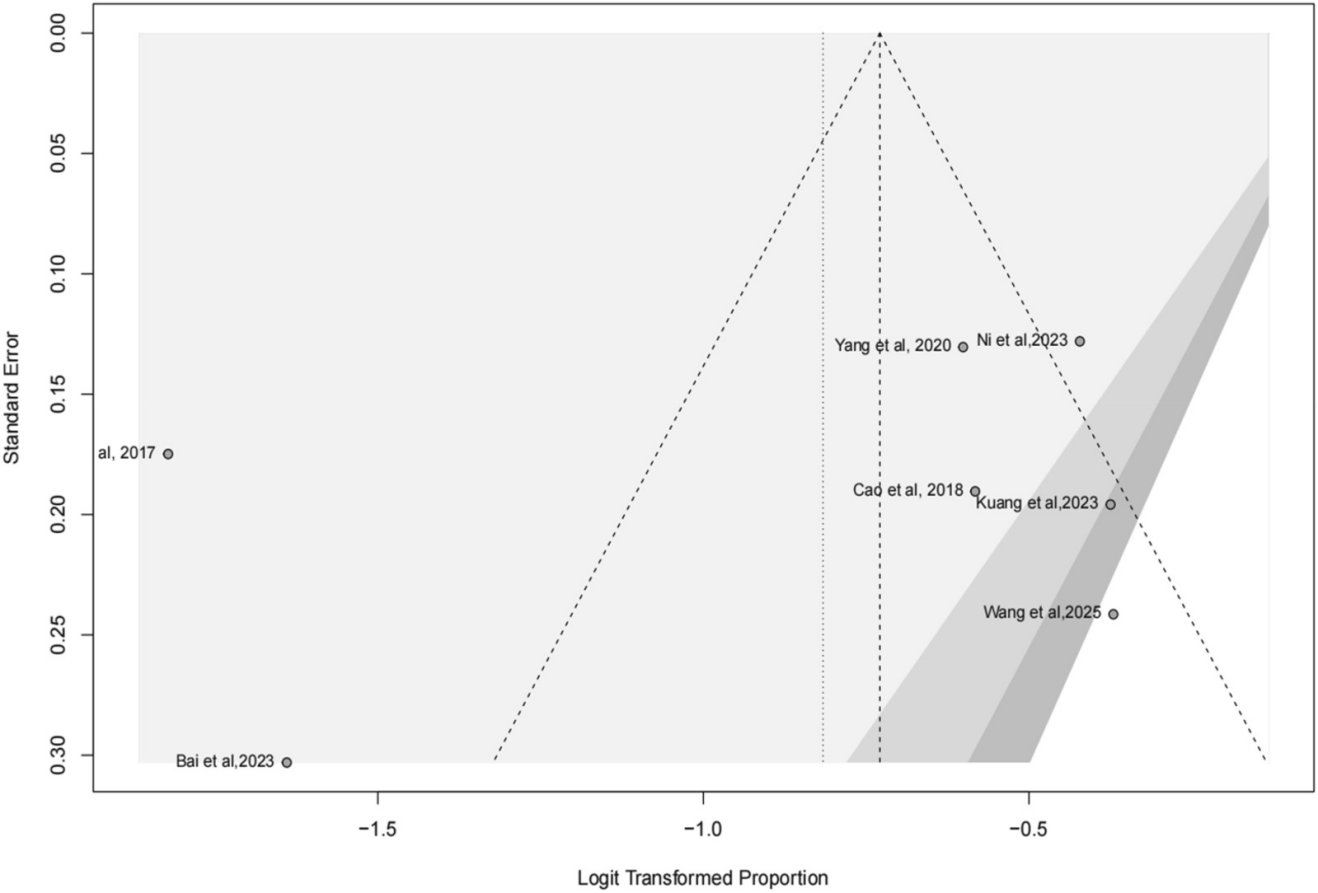
Standard funnel plot.

**FIGURE 4:**
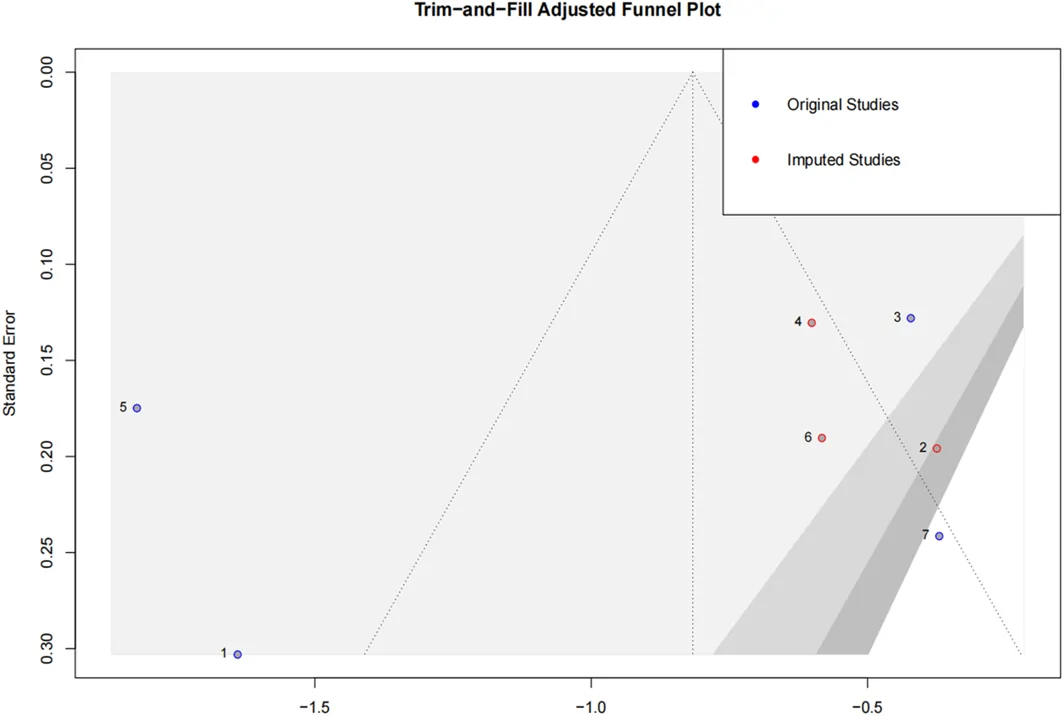
Trim‐and‐fill adjusted funnel plot.

The results of the funnel plot indicate that the literature on the prevalence of sarcopenia in patients with diabetic foot is asymmetrically distributed, suggesting a potential publication bias, as shown in Figure 3. In the Egger's test, *p* = 0.21, indicating a low likelihood of publication bias. After applying the trim‐and‐fill method, the inclusion of 3 virtual studies did not reverse the research results, with *k* = 0, and the combined effect remained stable (OR = −0.82, 95% CI: −1.27 to −0.36), with a prevalence rate of 30.7% before and after correction, suggesting that publication bias does not affect the stability of the research results, as shown in Figure 4.

#### 3.3.4 Effect Value Combination of Influencing Factors for Diabetic Foot Sarcopenia

In this study, factors reported in two or more studies were combined, and a total of four factors were included. Meta‐analysis results showed that longer diabetes duration was significantly associated with sarcopenia in patients with diabetic foot [OR = 3.33 (95% CI: 1.61–6.87), *I* ^2^ = 0, *p* > 0.05]. The specific results are shown in Table 4.

**TABLE 4:** Meta‐analysis results of influencing factors.

Influencing factors	Include studies	Meta‐analysis (random effect)		Meta‐analysis (fixed effect)		Heterogeneity test	
		OR (95% CI)	*p*	OR (95% CI)	*p*	*I* ^2^	*p*
Age [14, 15]	2	1.97 (0.50,7.74)	0.33	1.06 (1.01, 1.10)	0.01	89%	0.01
Long‐standing diabetes [11, 15]	2	3.33 (1.61,6.87)	0.01	3.33 (1.61, 6.87)	0.01	0%	0.60
HbA1c [11, 15]	2	2.48 (0.80,7.66)	0.12	1.73 (1.49, 2.02)	0.01	57%	0.13
TcPO2 [12, 15]	2	2.03 (0.35,11.73)	0.43	0.91 (0.85, 0.98)	0.01	90%	0.01

## 4 Discussions

### 4.1 Principal Findings and Context of the Existing Evidence

This meta‐analysis provides the first comprehensive quantitative synthesis, revealing a high prevalence of sarcopenia among patients with DFU. The current evidence base, though limited to 7 cross‐sectional studies predominantly from China, is characterized by moderate methodological quality and consistent use of multivariate analyses, providing a legitimate foundation for this review.

### 4.2 High Prevalence and Its Clinical Implications

Our pooled prevalence of 31% is substantially higher than the 23% reported in the general Asian type 2 diabetes population [22]. Crucially, our subgroup analysis unveiled a striking dose‐response relationship between ulcer severity (Wagner grade) and sarcopenia prevalence, escalating from 23% in grade 1%–2%–90% in grade 5. This powerful gradient suggests that the pathophysiological burden of the advanced wound is strongly linked to muscle wasting. However, the cross‐sectional nature of the data prevents definitive conclusions on causality; severe sarcopenia may predispose to worse ulcers, advanced ulcers may accelerate muscle loss, or both may share common underlying drivers.

### 4.3 In‐Depth Exploration of Heterogeneity: Methodological and Clinical Insights

The significant heterogeneity (*I* ^2^ = 90.1%) in the overall prevalence estimate was systematically investigated, with subgroup analyses identifying two primary sources. First, the choice of diagnostic criteria and tools introduced substantial methodological heterogeneity. Estimates derived from the Baumgartner criteria and DXA or CT were consistently higher (38%–41%) and homogeneous (*I* ^2^ = 0%), whereas the 2014 AWGS criteria, particularly when implemented with BIA [15], yielded a marked outlier (14%). As BIA is susceptible to fluid shifts common in DFU, we recommend prioritizing DXA in future research to improve reliability and comparability. Second, clinical severity, as measured by the Wagner grade, was a key clinical modifier, accounting for a significant portion of the variance, as previously detailed. Furthermore, in the most severe Wagner grade 5 cases, tissue loss and gangrene might artifactually inflate the prevalence of sarcopenia if muscle mass is measured in the affected limb, a potential confounding factor that future studies should address by measuring muscles at standard, non‐affected sites.

### 4.4 Spectrum of Associated Factors and Future Directions

Our quantitative synthesis identified longer diabetes duration as significantly associated with sarcopenia (OR = 3.33). However, a paramount limitation is that this conclusion rests exclusively on two studies [11, 15], rendering it preliminary. The pathophysiological link is plausible, involving mechanisms such as AGEs accumulation and chronic inflammation [23, 24, 25]. Nonetheless, the causal relationship remains to be established by prospective studies.

Qualitatively, we identified a spectrum of 15 other potential factors across patient, disease, and behavioral domains (e.g., age, HbA1c, and protein intake). The inability to pool these data quantitatively underscores the nascent stage of this field. Future large‐scale and prospective studies are required to confirm these associations and elucidate their relative contributions.

### 4.5 Limitations

Our study has several limitations. The inclusion of studies solely from China may affect the generalizability of our findings. The small number of studies for certain outcomes, particularly risk factors, limits the robustness of those conclusions. Furthermore, the exclusive use of cross‐sectional data precludes any causal inference. Finally, unmeasured variables such as specific inflammatory markers may serve as residual confounders.

## 5 Conclusion

Sarcopenia is highly prevalent among patients with diabetic foot ulcers, with its likelihood dramatically increasing with wound severity. Our analysis, based on only two studies, suggests that longer diabetes duration is associated with sarcopenia, but this finding must be interpreted with caution due to the limited evidence. These results advocate for further research to evaluate the potential benefits of sarcopenia screening in the comprehensive management of diabetic foot, which could pave the way for earlier interventions to mitigate muscle loss and improve patient outcomes.

## Author Contributions

**Wang Hui:** conceptualization (lead), methodology (lead), formal analysis (lead), software (lead), visualization (lead), writing – original draft preparation (lead), writing – review and editing (equal). **Yang Yaping:** investigation (equal), data curation (equal), writing – review and editing (supporting). **Lv Tan:** investigation (equal), data curation (equal), writing – review and editing (supporting). **Mao Zhigang:** validation (equal), investigation (supporting), writing – review and editing (supporting). **Dai Xinyi:** validation (supporting), resources (supporting), writing – review and editing (supporting). **Peng Yinbo:** validation (equal), investigation (supporting), resources (supporting), writing – review and editing (supporting). **Wang Jing:** conceptualization (lead), methodology (supporting), project administration (lead), supervision (lead), writing – review and editing (equal).

## Funding

The authors have nothing to report.

## Ethics Statement

This study is a systematic review and meta‐analysis of previously published studies. As such, ethical approval was not required for this work.

## Consent

The authors have nothing to report.

## Conflicts of Interest

The authors declare no conflicts of interest.

## Supporting information


Supporting Information S1

## Data Availability

All evidence was derived from previously published literature, and all relevant data are included within this article.
