# Supplementary material for: Prevalence and Associated Factors of Sarcopenia in Patients With Diabetic Foot Ulcers: A Systematic Review and Meta‐Analysis
Source: J Foot Ankle Res. 2026 Sep 16;19(3):e70201. doi: 10.1002/jfa2.70201 (PMC13583058; doi:10.1002/jfa2.70201)
Supplement: Supplementary file 1 [file JFA2-19-e70201-s001.docx]

**Table S1.** Search terms employed in the screening based on title, abstract, and keywords in the literature search.

| **Database** | **Search terms** |
| --- | --- |
|  |  |
| PubMed | (foot ulcer*[Title/Abstract] OR diabetic foot[Title/Abstract] OR foot disease*[Title/Abstract] OR diabetic foot ulcer*  [Title/Abstract] OR diabetic feet[Title/Abstract] OR diabetic foot[MeSH Terms]) AND (sarcopenia[Title/Abstract] OR  Muscular Atrophy[Title/Abstract] OR Muscle loss[Title/Abstract] OR skeletal muscle index[Title/Abstract] OR Muscle  attenuation[Title/Abstract] OR sarcopenia[MeSH Terms]) AND (risk factor* OR predictor* OR epidemiologic factor*  OR related factor* OR contributing factor* OR risk factors[MeSH Terms] OR prevalence OR morbidity rate) |
| Cochrane Library | (“foot ulcer” OR "diabetic foot" OR "foot disease" OR "diabetic foot ulcer" OR “diabetic feet” ) AND (skeletal muscle  index OR sarcopenia OR Muscular Atrophy OR Muscle attenuation OR Muscle loss ) AND（risk factor OR predictor  OR epidemiologic factor OR related factor OR contributing factor OR prevalence OR morbidity rate） |
| Web of Science | (“foot ulcer” OR "diabetic foot" OR "foot disease" OR "diabetic foot ulcer" OR “diabetic feet” ) AND (skeletal muscle  index OR sarcopenia OR Muscular Atrophy OR Muscle attenuation OR Muscle loss ) AND（risk factor OR predictor  OR epidemiologic factor OR related factor OR contributing factor OR prevalence OR morbidity rate） |
| Scopus | (“foot ulcer” OR "diabetic foot" OR "foot disease" OR "diabetic foot ulcer" OR “diabetic feet” ) AND (skeletal muscle  index OR sarcopenia OR Muscular Atrophy OR Muscle attenuation OR Muscle loss ) AND（risk factor OR predictor  OR epidemiologic factor OR related factor OR contributing factor OR prevalence OR morbidity rate） |
| Embase | (“foot ulcer” OR "diabetic foot" OR "foot disease" OR "diabetic foot ulcer" OR “diabetic feet” ) AND (skeletal muscle  index OR sarcopenia OR Muscular Atrophy OR Muscle attenuation OR Muscle loss ) AND（risk factor OR predictor  OR epidemiologic factor OR related factor OR contributing factor OR prevalence OR morbidity rate） |
| VIP | “糖尿病足”或“糖尿病溃疡”或“糖尿病足溃疡”或“足部疾病”或“糖尿病肢端坏疽”或“糖尿病神经病变”  或”糖尿病血管病变“和“骨骼肌指数”或“肌少症”或“肌肉萎缩”或“肌肉衰减”或“肌肉丢失”或“少肌  症”或”肌肉减少”或“骨骼肌衰减”或“骨骼肌减少” |
| SinoMed | “糖尿病足”或“糖尿病溃疡”或“糖尿病足溃疡”或“足部疾病”或“糖尿病肢端坏疽”或“糖尿病神经病变”  或”糖尿病血管病变“和“骨骼肌指数”或“肌少症”或“肌肉萎缩”或“肌肉衰减”或“肌肉丢失”或“少肌  症”或”肌肉减少”或“骨骼肌衰减”或“骨骼肌减少” |
| CNKI | “糖尿病足”或“糖尿病溃疡”或“糖尿病足溃疡”或“足部疾病”或“糖尿病肢端坏疽”或“糖尿病神经病变”  或”糖尿病血管病变“和“骨骼肌指数”或“肌少症”或“肌肉萎缩”或“肌肉衰减”或“肌肉丢失”或“少肌  症”或”肌肉减少”或“骨骼肌衰减”或“骨骼肌减少” |
| Wanfang | “糖尿病足”或“糖尿病溃疡”或“糖尿病足溃疡”或“足部疾病”或“糖尿病肢端坏疽”或“糖尿病神经病变”  或”糖尿病血管病变“和“骨骼肌指数”或“肌少症”或“肌肉萎缩”或“肌肉衰减”或“肌肉丢失”或“少肌  症”或”肌肉减少”或“骨骼肌衰减”或“骨骼肌减少” |
